# Significant differences in the degree of genomic DNA N^6^-methyladenine modifications in *Acidithiobacillus ferrooxidans* with two different culture substrates

**DOI:** 10.1371/journal.pone.0298204

**Published:** 2024-02-02

**Authors:** RuTao Lin, JingQi Liu, Si Shan, Yu Zhang, Yu Yang

**Affiliations:** 1 School of Minerals Processing and Bioengineering, Central South University, Changsha, Hunan, China; 2 Xiangya School of Medicine, Central South University, Changsha, Hunan, China; 3 School of Minerals Processing and Bioengineering, Key Laboratory of Biohydrometallurgy of the Ministry of Education, Central South University, Changsha, Hunan, China; The University of Akron, UNITED STATES

## Abstract

DNA N^6^-methyladenine (6mA) modification is widespread in organisms and plays an important functional role in the regulation of cellular processes. As a model organism in biohydrometallurgy, *Acidithiobacillus ferrooxidans* can obtain energy from the oxidation of ferrous iron (Fe^2+^) and various reduced inorganic sulfides (RISCs) under acidic conditions. To determine the linkage between genomic DNA methylation and the switching between the two oxidative metabolic pathways in *A*. *ferrooxidans*, the 6mA landscape in the genome of *A*. *ferrooxidans* cultured under different conditions was evaluated by using 6mA-IP-seq. A total of 214 and 47 high-confidence peaks of 6mA were identified under the Fe^2+^ and RISCs oxidizing conditions, respectively (P<10^−5^), suggesting that genomic methylation was greater under Fe^2+^ oxidizing conditions. 6mA experienced a decline at the transcription start site (TSS) and occurs frequently in gene bodies under both oxidizing conditions. Furthermore, Gene Ontology (GO) and Kyoto Encyclopedia of Genes and Genomes (KEGG) analyses revealed that 7 KEGG pathways were mapped into and most of the differentially methylated genes were enriched in oxidative phosphorylation and metabolic pathways. Fourteen genes were selected for studying the effect of differences in methylation on mRNA expression. Thirteen genes, excluding *petA-1*, demonstrated a decrease in mRNA expression as methylation levels increased. Overall, the 6mA methylation enrichment patterns are similar under two conditions but show differences in the enriched pathways. The phenomenon of upregulated gene methylation levels coupled with downregulated expression suggests a potential association between the regulation mechanisms of 6mA and the Fe^2+^ and RISCs oxidation pathways.

## 1 Introduction

*Acidithiobacillus ferrooxidans* is an acidophilic, aerobic and chemically autotrophic gram-negative bacterium that can inhabit natural environments where organic matter is scarce [1]. The bacterium can oxidize and utilize iron and reduced inorganic sulfides (RISCs) in minerals to obtain energy for growth and metabolism, which has an important effect on the geochemical cycle of iron, sulfur, and other elements in low-pH environments and is the driving force for ecological succession in acidic habitats [2]. In addition, the bacterium can accelerate the oxidation and dissolution of copper, nickel, cobalt, and other metal sulfide minerals, promote the formation of acid metal-rich drainage and facilitate the recovery of the above valuable metals from mineral leaching solutions. Meanwhile, it has been found that *A*. *ferrooxidans* can play an important role in the bioleaching of rare earth minerals. Its interesting physiological properties are also widely used in the bioleaching of heavy metals such as copper, nickel, cobalt and gold, as well as in the biodesulfurization of wastewater from the oil and coal industries [3].

*A*. *ferrooxidans* ATCC 23270 is a model strain of *A*. *ferrooxidans* whose genome has been fully sequenced and annotated, and two model oxidation pathways for *A*. *ferrooxidans* ATCC 23270, Fe^2+^ and RISCs, have been established in previous studies [4]. The coding genes and manipulators involved in these two oxidation pathways in *A*. *ferrooxidans* differ, and *A*. *ferrooxidans* regulates the expression of genes involved in the oxidation pathway according to the electron donor available in the environment. For example, the genes involved in Fe^2+^ oxidation are more highly expressed in the presence of Fe^2+^ than in the presence of RISCs, and vice versa [5]. *A*. *ferrooxidans* is more widely used in the bioleaching of chalcopyrite, spotted copper ores and some low-grade minerals [6]. The bioleaching rate of *A*. *ferrooxidans* during the leaching process for different typical sulfide ores differs significantly, and the expression level of each gene also differs greatly. Notably, when Fe^2+^ and RISCs coexist, Fe^2+^ is preferentially oxidized by *A*. *ferrooxidans*, while RISCs oxidation occurs after the complete oxidation of Fe^2+^ [7]. The above findings are supported by the results of gene expression profiling in *A*. *ferrooxidans*. Genes involved in Fe^2+^ oxidation are transcribed first, while genes involved in RISCs oxidation are transcribed after the complete oxidation of Fe^2+^. Presumably, there is a special regulatory mechanism in *A*. *ferrooxidans* that plays a key role in the utilization of both oxidation pathways. The inhibition of S metabolism during the actual leaching process causes the deposition of S monomers on the mineral surface and affects the continuous precipitation of metal ions, which limits the efficiency and applicability of *A*. *ferrooxidans* in mineral leaching.

Epigenetic modifications regulate gene expression and alter gene function without altering the DNA sequence. These modifications include DNA modifications, histone modifications, and transcription factor binding. In prokaryotes, DNA methyltransferase (MTase) transfers methyl groups from S-adenosyl methionine (SAM) to adenine or cytosine in DNA, forming three different modifications: 6-methyladenine (6-methyladenine, m6A), 4-methylcytosine (4-methylcytosine, m4C) or 5-methylcytosine (5-methylcytosine, m5C) [8]. Genomic DNA methylation is prevalent in all three domains of life and plays an important role in key cellular physiological processes in bacteria. For example, in *Escherichia coli*, the adenine MTase Dam plays an important role in DNA replication [9, 10]; in addition, the MTase CcrM of *Caulobacter crescentus* plays a key role in controlling the progression of the cell cycle [11]; and the m5C MTase plays an important role in orchestrating gene expression in *Helicobacter pylori* [12]. Furthermore, methylation has been shown to play a regulatory role in many physiological functions in bacteria [13–16], including in labeling promoter sequences, altering DNA stability and structure, and altering the affinity of DNA-binding proteins to affect gene expression [17, 18]. In addition, methylation affects gene expression by interfering with the separation of DNA strands. In conclusion, adenine methylation has been shown to play a more critical role in transcriptional regulation than other types of methylation [19].

*A*. *ferrooxidans* has been widely studied because of its special physiological characteristics and metabolic pathways, but many of its special physiological characteristics have still not been studied in depth [20]. Recent studies of genomic DNA methylation have shown that methylated DNA is widespread in organisms, and an increasing number of studies have identified its role in bacterial gene regulation and other processes, which has become an important direction for biological research [21, 22]. Studying the DNA methylation level of the bacterial genome is valuable not only for comprehending the fundamental biological characteristics of *A*. *ferrooxidans* adaptability but also for identifying new targets. It provides a theoretical foundation for the targeted modification of *A*. *ferrooxidans* through engineering, altering its priority for the utilization of Fe^2+^ and RISCs. This strategy is anticipated to achieve efficient biological oxidation of various refractory sulfide ores in a complex biometallurgical system where Fe^2+^ and RISCs coexist. Additionally, it is expected to enhance the efficient recycling of valuable metals.

## 2. Materials and methods

### 2.1 Strains, culture, and growth conditions

*A*.*ferrooxidans* ATCC 23270 was obtained from the Key Laboratory of Biometallurgy of the Ministry of Education, Central South University, Hunan, China. *A*. *ferrooxidans* was cultured under two different conditions: one group was cultured in 9K basal medium supplemented with FeSO_4_ (44.6 g/L), and the other group was cultured in medium supplemented with S^0^ (10.0 g/L, pH 2.0); the growth conditions were 30°C and 180 rpm [23]. To prepare growth curves, the seed solution was inoculated at 1% in the cultivation medium, and growth was measured every 12 hours for each condition. Three parallel experiments were performed for the two groups.

### 2.2 Determination of the specific growth rate μ

During the logarithmic growth period, the specific growth rate *μ* was calculated with the following equation [24]:

$$\mu =\frac{1}{X}∙\frac{dX}{dt}$$ (1)

The specific growth rate between *t*_*1*_ and *t*_*2*_ was calculated as follows:

$$\int_{t1}^{t_{2}}\mu dt=\int_{X_{1}}^{X_{2}}\frac{1}{X}dX$$ (2)

$$\mu (t_{2}-t_{1})=lnX_{2}-lnX_{1}$$ (3)

$$\mu =\frac{lnX_{2}-lnX_{1}}{t_{2}-t_{1}}$$ (4)

where *dX* is the increase in biomass, *dt* is the process time, *X* is the biomass, and *X*_*1*_ and *X*_*2*_ are the biomasses at *t*_*1*_ and *t*_*2*_, respectively.

### 2.3 DNA extraction and fragmentation

*A*. *ferrooxidans* was cultured to the logarithmic phase under two different conditions, and the genomic DNA (gDNA) of *A*. *ferrooxidans* was extracted using a Bacterial DNA Extraction Kit (TIANGEN, China) following the manufacturer’s protocol. To exclude contaminating RNA, the gDNA was treated with RNase A. The quality and concentration of the DNA samples were measured using an ND-100C Ultra-Micro UV-visible spectrophotometer (MiuLab, China). Approximately 6 μg of each gDNA sample was sonicated to obtain fragments of approximately 200 bp by using an ultrasonic crusher, and the fragments were then purified. The extracted gDNA samples from *A*. *ferrooxidans* were broken into fragments of 200 bp using an ultrasonic crusher as follows: 130 W power at 30% and 50 kHz, with 3 s on and 5 s off; this process was repeated multiple times for a total of 8 minutes. gDNA fragments were repaired end-to-end by adding adenine using the Fast DNA End Repair Kit (Thermo Fisher Scientific, USA). The linked DNA fragments were then denatured and immunoprecipitated with 3 mg of 6mA antibody (Synaptic Systems, Germany). The bound DNA was treated with protease K and purified.

### 2.4 6mA IP-Seq and bioinformatic analyses

For 6mA-IP-seq, four libraries (immune-precipitated DNA and their input) were prepared according to the manufacturer’s instructions, and the DNA samples were sequenced on an Illumina NovaSeq 6000 platform (Illumina, USA) with 200 bp paired-end reads. The raw data were trimmed using Solexa pipeline software v 1.8 (off-line base caller software, v 1.8) and checked with FastQC (v 0.11.7) [25]. Reads with N values greater than 10% or lower than 10 for 26 DNA samples were sequenced on an Illumina NovaSeq 6000 platform (Illumina, USA) with 200 bp paired-end reads. The raw data were trimmed using Solexa pipeline software v 1.8 (Off-Line Base Caller software, v 1.8) and checked with FastQC (v 0.11.7). Reads with more than 10% N bases or more than 50% low-quality bases were filtered out using Trimmomatic (v 0.32). The high-quality reads were selected for analysis, and repeated reads were deleted by SAMtools (v 1.9) [26, 27]. Afterwards, a series of analyses were performed by identifying 6mA-enriched region peaks, including 6mA level difference analysis, and heatmaps were generated [28]. The potential functions of the genes differentially methylated under two different oxidizing conditions were predicted by Gene Ontology (GO) (http://www.geneontology.org, accessed on 26 December 2023) and Kyoto Encyclopedia of Genes and Genomes (KEGG) (http://www.genome.jp/kegg/, accessed on 26 December 2023) pathway enrichment analyses [29].

### 2.5 Total RNA extraction

*A*. *ferrooxidans* was cultured to the logarithmic phase under Fe^2+^ and RISCs oxidizing conditions. The bacterial solution was collected by centrifugation at 9000 rpm for 10 minutes and thoroughly cleaned twice with PBS, after which the supernatant was discarded by centrifugation. The bacterial RNA was extracted with TRIzol Reagent (Vazyme, China) following the manufacturer’s instructions. The RNA samples were treated with gDNA wiper mix (Vazyme, China) to remove residual genomic DNA contamination. Total bacterial RNA was quantified at OD_260_ and OD_280_ with an ND-100C Ultra-Micro UV‒visible spectrophotometer (MiuLab, China). Three replicates of purified RNA were examined for each sample.

### 2.6 Reverse transcription quantitative polymerase chain reaction analysis

To verify the results, fourteen genes with different degrees of methylation under the two oxidizing conditions were selected for real-time quantitative reverse transcription polymerase chain reaction (RT–PCR) analysis. The primers used were designed on the National Center for Biotechnology Information (NCBI) website (https://www.ncbi.nlm.nih.gov/, accessed on 26 December 2023), evaluated by Primer Premier 5.0 software, and synthesized by Tsingke Biotechnology (Beijing) Co., Ltd. All the primer pairs used in this study are listed in S1 Table. The results were normalized against a control gene (16S rRNA) to correct for sample-to-sample variation. The RNA was reverse-transcribed with a HiScript® Ⅱ Q RT SuperMix for qPCR Kit (Vazyme, China). The 20 μL reactions were performed with 40 cycles of 10 s at 95°C, 30 s at 55°C, and 60 s at 72°C and monitored in a Q2000B real-time PCR detection system (Long Gene Instruments, China) using ChamQ Universal SYBR qPCR Master Mix (Vazyme, China). The expression of each gene was determined from three replicates of a single RT‒PCR experiment. PCR products were assessed by melting curve analysis, and the ratios of the gene expression levels under different treatments were calculated from the ratios of signal intensities between the two different oxidizing conditions and normalized to the 16S rRNA gene expression level and dilution factor.

### 2.7 Statistical analysis

All the experiments were repeated at least three times. All the data are expressed as the mean ± standard deviation unless otherwise stated. SPSS 20.0 software was used for relevant statistical analysis. *p < 0.05, **p < 0.01, or ***p < 0.001 was considered to indicate statistical significance.

## 3. Results and discussion

### 3.1 Growth of A. ferrooxidans on two different substrates (Fe^2+^ and RISCs)

**Fig 1:**
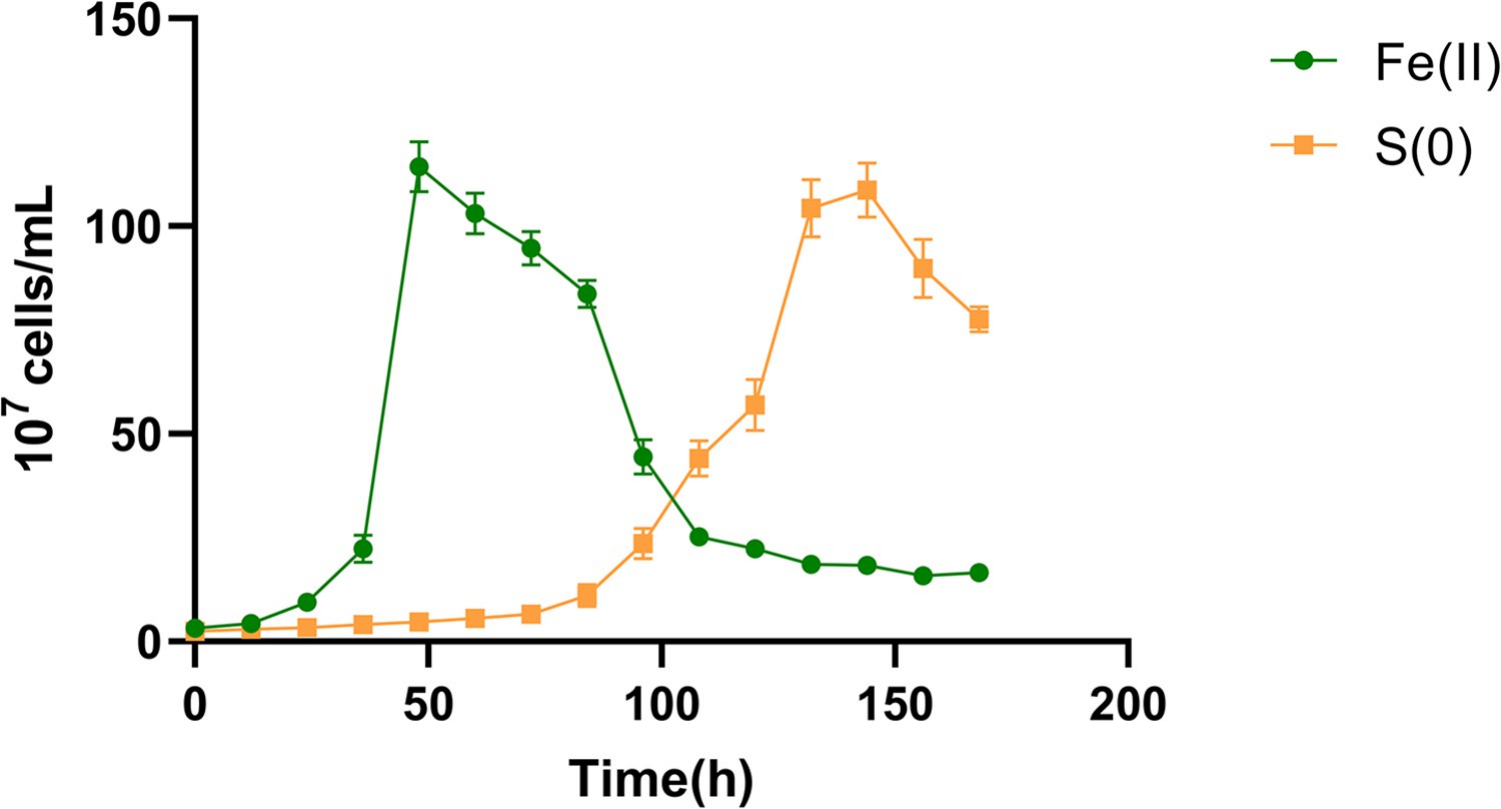
Growth curves of *A*. *ferrooxidans* under Fe^2+^ and RISCs oxidizing conditions.

*A*. *ferrooxidans* could grow normally on both substrates, but the growth delay period was different (Fig 1). When cultured under the Fe^2+^ oxidizing conditions, the lag phase was shorter, with cells entering the logarithmic growth period after approximately 20 hours, while under RISCs oxidizing conditions, the lag period was nearly 90 hours. Based on the results of the experiment, the optimal times for extracting genomic DNA from *A*. *ferrooxidans* ATCC 23270 under the Fe^2+^ and RISCs oxidizing conditions were 48 hours and 120 hours after the start of cultivation, respectively. At these times, the bacteria were in the logarithmic growth phase and exhibited strong bacterial activity. The maximum specific growth rate (*μ*_max_) in the log phase was calculated; in Fe^2+^ oxidizing conditions, *μ*_max_ = 0.14, while in RISCs oxidizing conditions, *μ*_max_ = 0.06. The cells exhibit a faster specific growth rate and a shorter delay period when Fe is used in the energy mechanism than when S is used in the energy mechanism, even though the same molar mass of S provides more electrons and produces more ATP [30]. The hydrophobicity of S monomers may hinder the utilization of S by *A*. *ferrooxidans*, which can be improved by the addition of surfactants without affecting the utilization of Fe [31].

However, eventually, the biomass under the RISCs oxidizing conditions was not higher than that under the Fe^2+^ oxidizing conditions. On the one hand, mineral depletion in the medium may be responsible for this difference; on the other hand, cell lysis might be responsible for limiting biomass under Fe^2+^ oxidizing conditions and may be responsible for the decline in biomass after the logarithmic period [32]. *A*. *ferrooxidans* prefers Fe^2+^ as an energy resource when growing. *A*. *ferrooxidans* begins to assimilate RISCs until Fe^2+^ is depleted [33]. This process is very unfavorable for leaching ores with high sulfur and low iron contents. However, these results suggested that iron-metabolizing genes may be expressed at higher levels than sulfur-metabolizing genes. In conclusion, *A*. *ferrooxidans* was able to grow with Fe and S as the only energy substrates, despite having different utilization efficiencies for the two energy substrates.

### 3.2 The degree of N^6^-methyladenine modification in A. ferrooxidans ATCC 23270 cultured with two substrates

**Fig 2:**
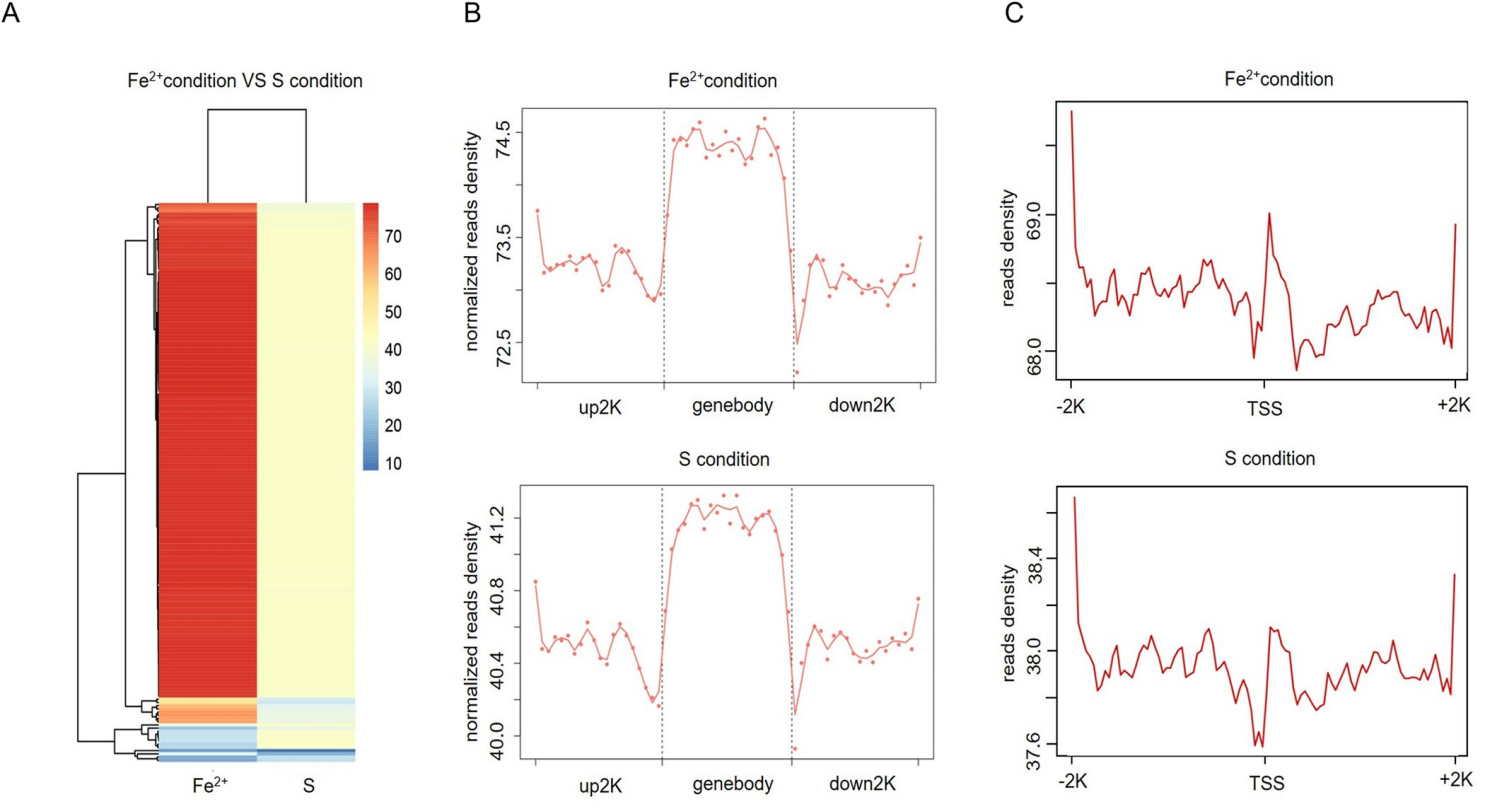
Distribution pattern of 6mA in the genomic DNA of *A*. *ferrooxidans* ATCC 23270. (A) Heatmap of 6mA modifications in the genomic DNA of *A*. *ferrooxidans* ATCC 23270 under Fe^2+^ and RISCs oxidizing conditions. The genome methylation in the Fe^2+^ oxidizing conditions was mostly upregulated (in red) and, to a small extent, downregulated (in blue) compared to that in the RISCs oxidizing condition, while the genes within the group differed more. (B) Distribution pattern of 6mA peaks around the gene body in *A*. *ferrooxidans* ATCC 23270 under Fe^2+^ (top panel) and RISCs (bottom panel) oxidizing conditions. 6mA levels were greater in gene bodies than in intergenic regions under both conditions. (C) Distribution pattern of 6mA peaks around the TSS in *A*. *ferrooxidans* ATCC 23270 under Fe^2+^ (top panel) and RISCs (bottom panel) oxidizing conditions. 6mA levels decreased at the TSS, followed by a sharp increase immediately after the TSS under both conditions.

The treated gDNA fragments were analyzed by identifying 6mA methylation-enriched region peaks for 6mA level difference analysis and to obtain a heatmap (Fig 2A). The heatmap shows that the genomes of *A*. *ferrooxidans* ATCC 23270 cultured on two different substrates were significantly different between the two groups of samples, indicating a greater difference in the degree of genome methylation.

Under the Fe^2+^ and RISCs oxidizing conditions, 214 and 47 high-confidence peaks of 6mA, respectively, were identified (P<10^−5^). Approximately 20% of the peaks were common under the two conditions, suggesting that 6mA was not only associated with the regulation of genes involved in the utilization of both energy substrates but also participated in a maintenance mechanism at specific genomic regions in *A*. *ferrooxidans* ATCC 23270. Significantly, the majority of the gene-specific 6mA peaks under the Fe^2+^ oxidizing conditions were wider than those under the RISCs oxidizing conditions, and several of them consisted of multiple subpeaks (S1 Fig). This result suggested that the 6mA sites were more enriched in these regions in response to Fe^2+^ culture, which was corroborated by the heatmap in Fig 2A. In addition, under both culture oxidizing conditions, the 6mA levels were greater in the gene bodies than in the intergenic regions (Fig 2B), and the 6mA levels decreased at the TSS, followed immediately by a sharp increase (Fig 2C). The TSS plot pattern, where 6mA was depleted from TSSs, was encountered in rice and mice [34, 35]. However, this process is different from that in green algae and fungi [36, 37], in which 6mA sites are enriched around the TSSs. While the distribution patterns of 6mA around TSSs in rice and green algae differ, research has demonstrated that their respective distribution patterns contribute to nucleosome positioning and enhanced gene expression. These findings indicated that the distribution patterns of 6mA around TSSs are associated with gene expression. Based on the similar distribution patterns between *A*. *ferrooxidans* and rice, it was postulated that this association also existed in the former. Currently, there is limited research on the distribution patterns of 6mA around TSSs in bacteria, and further investigation of the underlying mechanisms is needed. Overall, the Fe^2+^ oxidizing conditions produced higher methylation levels than the RISCs oxidizing condition, but methylation still exhibited a similar pattern under both oxidizing conditions: concentrated enrichment in the gene body region and a decrease in the TSS region.

### 3.3 GO and KEGG analyses revealed that the upregulated and downregulated genes were significantly enriched in different functional regions

The genes that differed between the two substrates were divided into two broad categories: those with increased methylation in the Fe^2+^ oxidizing condition compared to the RISCs oxidizing condition and those with decreased methylation in the Fe^2+^ oxidizing condition compared to the RISCs oxidizing condition (S2 Table). GO and KEGG analyses of the genes with different degrees of methylation on the two substrates were also conducted to predict the functions and molecular interactions among the genes.

**Fig 3:**
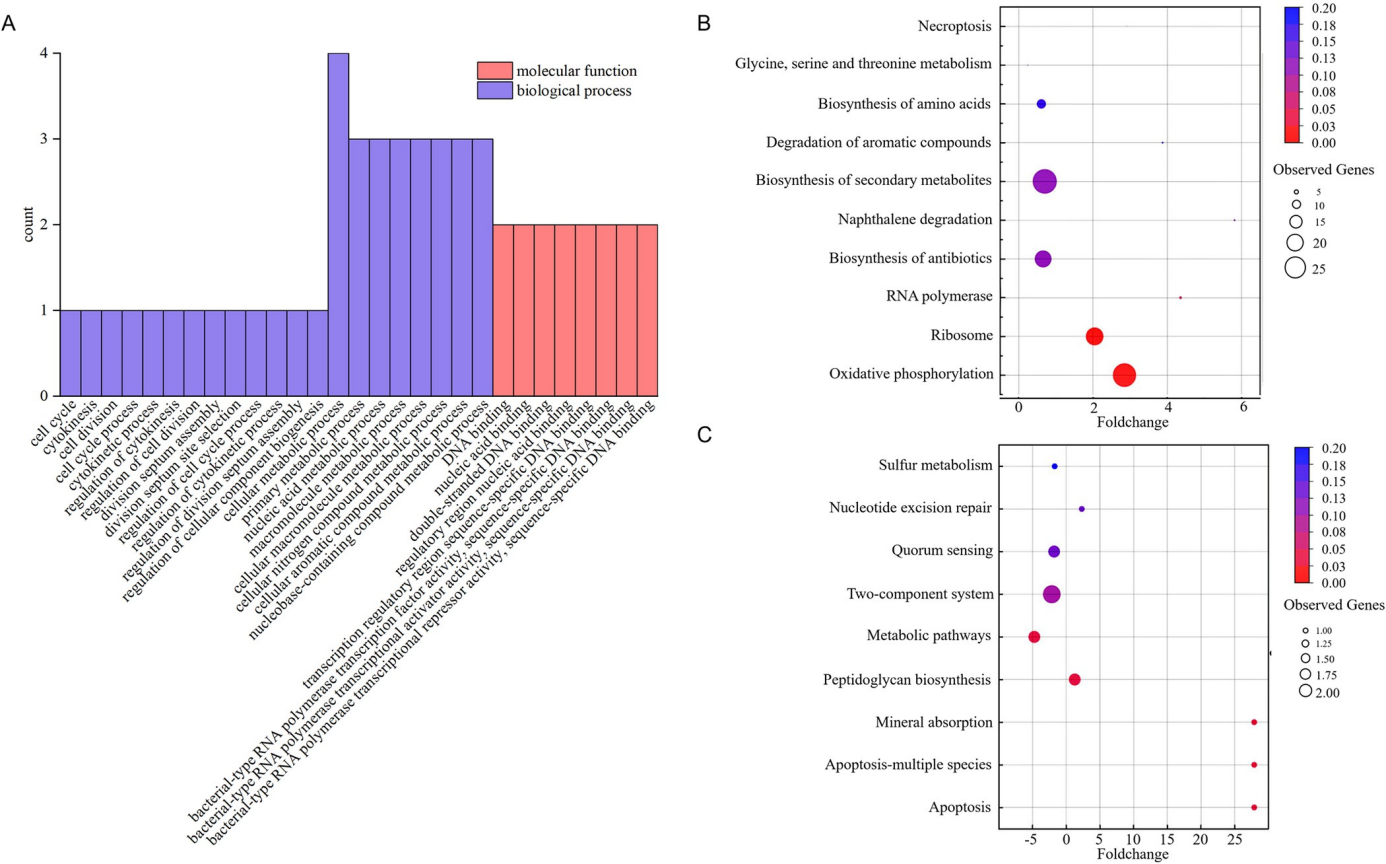
GO and KEGG functional enrichment analyses of the differentially methylated genes. (A) Top 21 significant Gene Ontology (GO) terms of differentially methylated genes for *A*. *ferrooxidans* under Fe^2+^ and RISCs oxidizing conditions. (B and C) KEGG pathway enrichment analysis of hypermethylated (B) and hypomethylated genes (C) of *A*. *ferrooxidans* under Fe^2+^ and RISCs oxidizing conditions. Hypermethylated genes are genes with an increased degree of methylation in the Fe^2+^ oxidizing condition compared with the RISCs oxidizing condition, and hypomethylated genes are genes with a decreased degree of methylation in the Fe^2+^ oxidizing condition compared with the RISCs oxidizing condition. The fold change represents the degree of enrichment of the differentially expressed genes. The Y-axis shows the names of the enriched pathways. The area of each node represents the number of observed genes. The significance test is represented by a color scale.

In general, GO analyses cover three domains: biological processes, cellular components, and molecular function. However, GO analysis of the genes in this study revealed enrichment in only two categories: biological processes, which contained 21 subcategories, and molecular function, which contained 8 subcategories. The significantly enriched GO terms with p < 0.1 are shown in Fig 3A. The genes were mainly involved in DNA binding, cellular metabolic processes, RNA polymerase transcription, cell cycle control, cell division, and cytokinesis. The results suggested that some genes exhibiting differential methylation levels between the two matrices were associated with cellular proliferation. It is well known that DNA methylation in most bacterial genomes plays an important role in DNA replication and mismatch repair [22], which was consistent with the results of the above GO analysis.

Based on the KEGG classification, the genes with different degrees of methylation in two substrates were mapped into 7 KEGG pathways (p< 0.05). Two main pathways were found to be enriched for the upregulated genes: the oxidative phosphorylation pathway and the ribosome pathway (Fig 3B). For the downregulated genes, the most enriched pathways were metabolic pathways, peptidoglycan biosynthesis, mineral absorption, and apoptosis (Fig 3C). By comparative analysis, three subunits associated with ATP synthase, *atpB*, *atpC* and *AFE_RS09445*, and six subunits of quinone oxidoreductase, *nuoD*, *nuoE*, *nuol*, *nuoJ*, *nuoK*, and *nuoL*, were methylated under Fe^2+^ oxidizing conditions, revealing differences compared to RISCs oxidizing conditions. Possibly because of the difference in energy density observed between Fe and S, the RISCs metabolic pathway, which provides more ATP, caused differences in methylation enrichment among the oxidative phosphorylation-related genes. In contrast, under S oxidizing conditions, the gene *AFE_RS09245*, which encodes an acyl-homoserine-lactone (AHL) synthase that is part of the AI-1 quorum sensing (QS) system in *A*. *ferrooxidans*, is methylated. This gene stimulates the synthesis of biofilms and is more highly expressed under Fe^2+^ oxidizing conditions [38]. This may also relate to the methylation of *murE*, which encodes the enzyme that catalyzes peptidoglycan synthesis, under the same conditions.

A comparison of the methylation enrichment levels under different conditions revealed that the difference in methylation in *A*. *ferrooxidans* was correlated with the Fe^2+^ and RISCs oxidation pathways.

### 3.4 The expression levels of genes with different N^6^-methyladenine modifications

**Fig 4:**
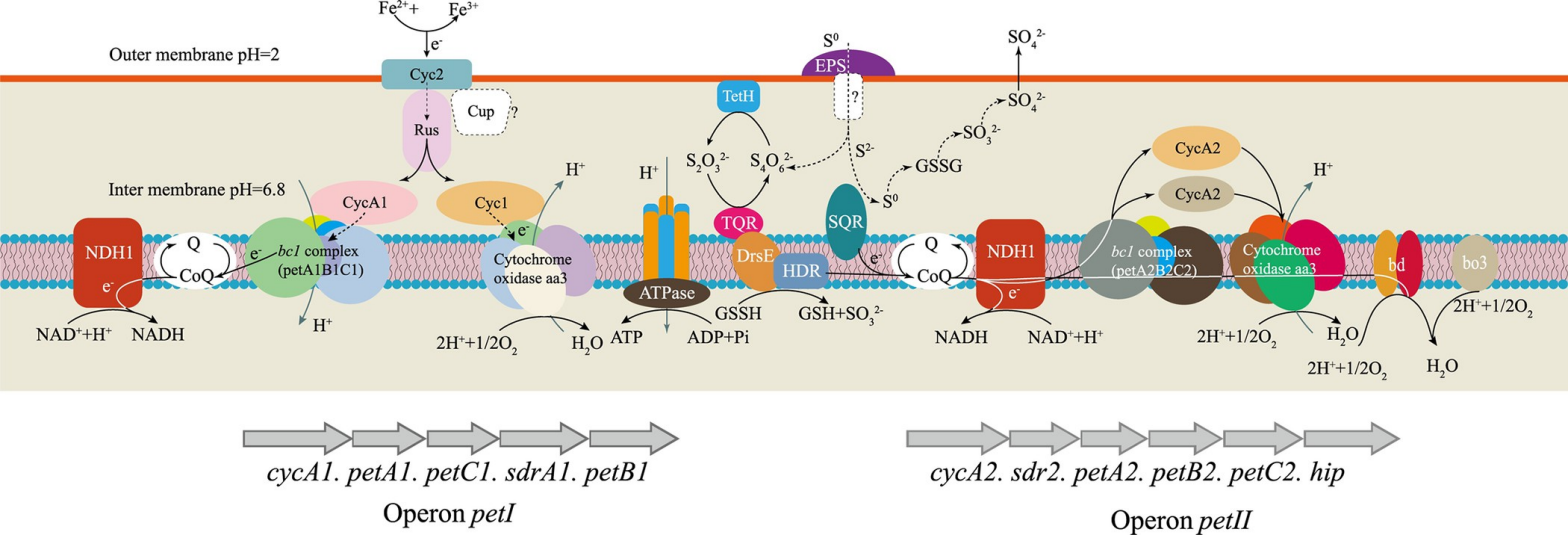
Model diagram of ferrous iron and the sulfur oxidation pathway in *A*. *ferrooxidans*.

**Fig 5:**
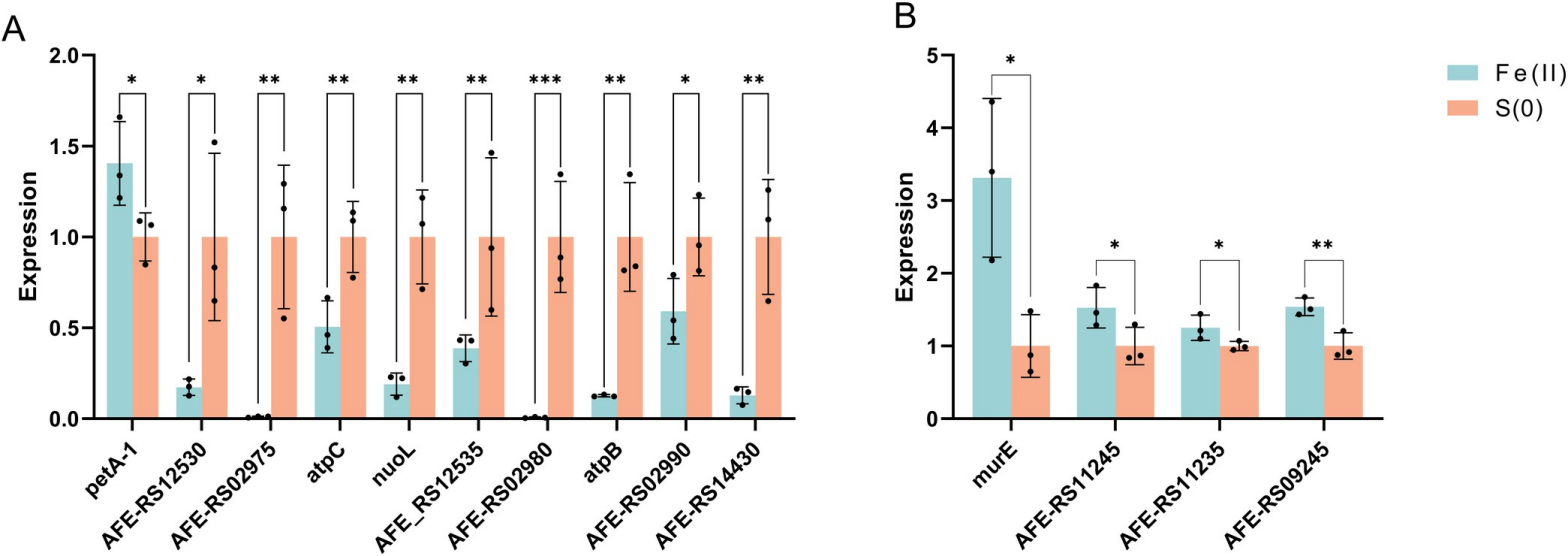
Relative expression levels of genes with different degrees of methylation. (A and B) The relative expression levels of genes with upregulated (A) and downregulated (B) methylation in Fe^2+^ oxidizing conditions compared to those in RISCs oxidizing conditions.

The RT‒PCR results for fourteen genes with different methylation levels identified under two different oxidizing conditions showed that the relative expression of nine of the ten genes decreased significantly with increasing methylation level, and the gene with the most significant difference in expression was *petA-1*, which plays a crucial role in the oxidative metabolism of Fe^2+^ in *A*. *ferrooxidans* (Fig 4). While the relative expression levels of the other four selected genes increased with decreasing methylation levels (as shown in Fig 5A and 5B), not all the genes with increased methylation showed decreased expression, and *petA-1*, an important component of the key manipulator *pet I* in the ferrous iron oxidation pathway, was significantly upregulated when the cells were cultured under Fe^2+^ oxidizing conditions. Apparently, DNA methylation was usually used as a transcriptional repressor signal in previous studies, and promoter methylation is usually associated with gene repression; however, recent studies have shown that DNA methylation can also activate gene expression, but the pathway and principle for methylation-stimulated gene transcription are not currently known [39].

Overall, the methylation of most genes was negatively correlated with the degree of expression, and iron-sulfur-related genes were intensively methylated under different conditions. The behavior of DNA methyltransferases also seems to be influenced by Fe^2+^ and RISCs oxidation conditions, which results in the methylation of related metabolic genes. In a previous study, the expression of two ferrous oxide-manipulating proteins, *petI* and *petII*, which are associated with iron–sulfur metabolism, was affected by Fe^2+^ and RISCs oxidation conditions [5]. However, *petI* responds to ferrous regulation, but the potential binding site ferric uptake regulator (FUR) box for iron-regulated proteins has not been identified [40]. Regarding the *petII* manipulator, the sequence TAAAATGAGATTGATAGTT was identified in its promoter region as a potential FUR box (S2 Fig). Other studies have shown that the AFE2726 protein, a protein responsive to S-regulation, is involved in the binding of the *petII* manipulator [41]. Methyltransferases may be affected by the same mechanism and are regulated by Fe^2+^ and RISCs induction. In other species, enrichment of methylation downregulates DNA expression, and *A*. *ferrooxidans* in the present study exhibited a similar result, where methylation may be one of the reasons for the downregulation of gene expression. However, *petA-1*, a *petI* gene deeply involved in iron metabolism [42], seems to be upregulated due to increased methylation. The expression of *petA-1*, which is part of the *petI* gene cluster, may be upregulated due to regulatory coupling with other genes in the cluster, or it may be due to methylation of different loci as a result of the involvement of different types of transferases in this regulation; however, this aspect still needs to be further investigated. The reduced expression of methylation-enriched genes provides new insights into the regulatory mechanisms in *A*. *ferrooxidans*, wherein methylation of the corresponding RISCs metabolism genes is inhibited, leading to enhanced utilization of S in the presence of Fe (II), e.g., leaching of high-sulfur minerals or the deposition of S monomers during the leaching process, thus improving the efficiency of metal leaching. In recent years, the modification of methyltransferase activity by the addition of methyltransferase inhibitors to modulate epigenetic inheritance has become a research priority, especially in the field of cancer research [43–45]. The high level of genetic manipulation of *A*. *ferrooxidans* associated with gene editing has become an obstacle to *A*. *ferrooxidans* regulation [20], although reports of successful construction of *A*. *ferrooxidans* mutants have emerged in recent years. If methylation regulation is achieved, the preference for iron–sulfur utilization can be flexibly changed to adapt to the different needs of different application scenarios, ensuring the integrity of the autologous genome of *A*. *ferrooxidans* while reducing the difficulty of regulatory manipulation.

## 4. Conclusion

This work investigated the extent of 6mA methylation in *A*. *ferrooxidans* genomic DNA under Fe^2+^ and RISCs oxidizing conditions. The results showed that there were significant differences in methylation between the two oxidizing conditions, and the methylation sites were concentrated at oxidative phosphorylation sites under Fe^2+^ oxidizing conditions relative to RISCs oxidizing conditions. Fe^2+^ and RISCs metabolism genes were found to be downregulated by methylation, except for *petA-1*, as determined by RT‒PCR, indicating that this methyltransferase is involved in the regulatory transformation of iron–sulfur metabolism, reflecting the complexity of the regulatory mechanism and the diversity of these genes. In addition, due to the upregulation of *petA-1* expression, we speculate that certain 6mA methylation sites may be able to achieve gene overexpression, but the reasons underlying the overexpression still need to be further investigated. The present study provides a reference for in-depth study of the transformation of Fe^2+^ and the RISCs oxidation pathway in *A*. *ferrooxidans* and is expected to reveal the mechanism of the efficient oxidation of ore under complex bioleaching conditions to improve the level of bioleaching and to realize the efficient recycling of valuable metals.

## Supporting information

S1 Table Partially methylated genes with different degrees of methylation and their primers. (PDF)

S2 Table Methylated differentially expressed genes in enrichment pathways. The symbol “+” refers to genes with an increased degree of methylation in one condition compared with the other condition, and the symbol “-” refers to genes with a decreased degree of methylation.
(PDF)

S1 Fig Snapshot of the 6mA peak determined by 6mA-IP-seq in the genomic DNA of *A*. *ferrooxidans* ATCC 23270 under Fe^2+^ and RISCs oxidizing conditions. (TIF)

S2 Fig Schematic representation of the *petII* operon promoter. The -10 region, -35 region, and possible fur box are labeled with blue, green, and yellow boxes, respectively.
(TIF)
