# Supplementary material for: Significant differences in the degree of genomic DNA N6-methyladenine modifications in Acidithiobacillus ferrooxidans with two different culture substrates
Source: PLoS One. 2024 Feb 2;19(2):e0298204. doi: 10.1371/journal.pone.0298204 (PMC10836689; doi:10.1371/journal.pone.0298204)
Supplement: S1 Table — (PDF) [file pone.0298204.s001.pdf]

Table S1 Partially methylated genes with different degrees of methylation and their primers

Gene ID	Sequence		Product size(bp)	Tm (°C)
	Forward primer (5' –3')	Reverse primer (5' –3')		
atpB	CCAGCGTCGTATTCAGGTT	GAGGGAGTGGTGGATTTCAT	214	55.5
atpC	TTCCGCTTGAATGTTGTCG	GCTGGGTAATGCGTAACTCG	154	56.8
murE	GCTTTGCCAGACGGGATG	TGCTCTATGTCGGACTGAACAC	143	56.2
nuoL	TGGAAGACCAGGAAAAACATC	CTCATTATCGAGGCAGTAGGG	122	54.7
petA-1	CCGTGGCAGAAGAAACCG	TCCCGACATACCATTCAGGAAC	172	52.7
AFE-RS02975	GCTTACCCATTCTCCTGTGC	GCCATACCAGGGCGAAAC	203	54.4
AFE-RS02980	GTTTCTGGCACTGGACAT	CACCACCTCAGTAGTAAAGC	190	52
AFE-RS02990	GCCATGACATTGATGTGCG	GTGCTGGGCTTGAGGTAGAG	128	56.8
AFE-RS09245	AGAACGGGATGGAGTCGG	CGGGAGAACATGGTGGTG	174	56.3
AFE-RS11235	TGACCTGCGGACATTGCG	TGGCGTGAGGGTGACATCG	86	56.4
AFE-RS11245	TCAGGCGTTTGGGTCTTG	ATCATCGGCATGTGTGGC	106	55.6
AFE_RS12530	CCATACCCAACAAGACTGCC	AACGCCCCCAATCTTTCC	220	54.4
AFE_RS12535	CATGTCCAGAGCCGATGT	GTCCTGACCCTTGTAGCG	157	53.1
AFE-RS14430	GCCACAAATCAAGGAACAC	ATCCCAAGCAGGGCGTAA	230	52.7
16s	TTAGTTGCCAGCGGTTCG	GGCTTGGCTTCCCTCTGTAC	145	55.6
