# Supplementary material for: Significant differences in the degree of genomic DNA N6-methyladenine modifications in Acidithiobacillus ferrooxidans with two different culture substrates
Source: PLoS One. 2024 Feb 2;19(2):e0298204. doi: 10.1371/journal.pone.0298204 (PMC10836689; doi:10.1371/journal.pone.0298204)
Supplement: S2 Table — The symbol “+” refers to genes with an increased degree of methylation in one condition compared with the other condition, and the symbol “-” refers to genes with a decreased degree of methylation. (PDF) [file pone.0298204.s002.pdf]

Table S2 Methylated differentially expressed genes in enrichment pathways

Term Name	GeneID	Description	Degree of gene methylation	
			Fe(II)	S(0)
**Oxidative phosphorylation**	*atpB*	F0F1 ATP synthase subunit A	+	-
	atpC	ATP synthase F1 subunit epsilon	+	-
	nuoD	NADH-quinone oxidoreductase, D subunit	+	-
	nuoE	NADH-quinone oxidoreductase, E subunit	+	-
	nuoI	NADH-quinone oxidoreductase, I subunit	+	-
	nuoJ	NADH-quinone oxidoreductase, J subunit	+	-
	nuoK	NADH-quinone oxidoreductase, K subunit	+	-
	nuoL	NADH-quinone oxidoreductase, L subunit	+	-
	petA-1	ubiquinol—cytochrome c reductase, iron-sulfur subunit	+	-
	AFE_RS12530	ubiquinol—cytochrome c reductase, cytochrome b subunit	+	-
	AFE_RS12535	ubiquinol—cytochrome c reductase, cytochrome c1 subunit	+	-
	ppa	inorganic pyrophosphatase	+	-
	AFE_RS02970	ubiquinol oxidase subunit II	+	-
	AFE_RS02975	cytochrome o ubiquinol oxidase, subunit I	+	-
	AFE_RS02980	cytochrome c oxidase subunit 3	+	-
	AFE_RS02985	cytochrome o ubiquinol oxidase	+	-
	AFE_RS02990	heme o synthase	+	-
	AFE_RS04510	complex I NDUFA9 subunit family protein	+	-
	AFE_RS07710	NADH dehydrogenase, putative	+	-
	AFE_RS09445	F0F1 ATP synthase subunit gamma	+	-
	AFE_RS14250	cytochrome b N-terminal domain-containing protein	+	-
	AFE_RS14255	cytochrome c1	+	-
	AFE_RS14420	hypothetical protein	+	-
	AFE_RS14425	cytochrome c oxidase, aa3-type, subunit I	+	-
	AFE_RS14430	cytochrome c oxidase, aa3-type, subunit II	+	-
**Apoptosis–multiple species**	*AFE_RS11245*	cytochrome c family protein	-	+
**Peptidoglycan biosynthesis**	*AFE_RS01000*	penicillin-binding protein 2	-	+
	murE	UDP-N-acetylmuramoylalanyl-D-glutamate—2,6-diaminopimelate ligase	-	+
**Mineral absorption**	*AFE_RS11235*	heavy metal-binding protein, putative	-	+
**Metabolic pathways**	*AFE_RS09245*	acyl-homoserine-lactone synthase	-	+

The symbol "+" refers to genes with an increased degree of methylation in one condition compared with the other condition, and the symbol "-" refers to genes with a decreased degree of methylation.
