# Supplementary figures and images for: Significant differences in the degree of genomic DNA N6-methyladenine modifications in Acidithiobacillus ferrooxidans with two different culture substrates
Source: PLoS One. 2024 Feb 2;19(2):e0298204. doi: 10.1371/journal.pone.0298204 (PMC10836689; doi:10.1371/journal.pone.0298204)

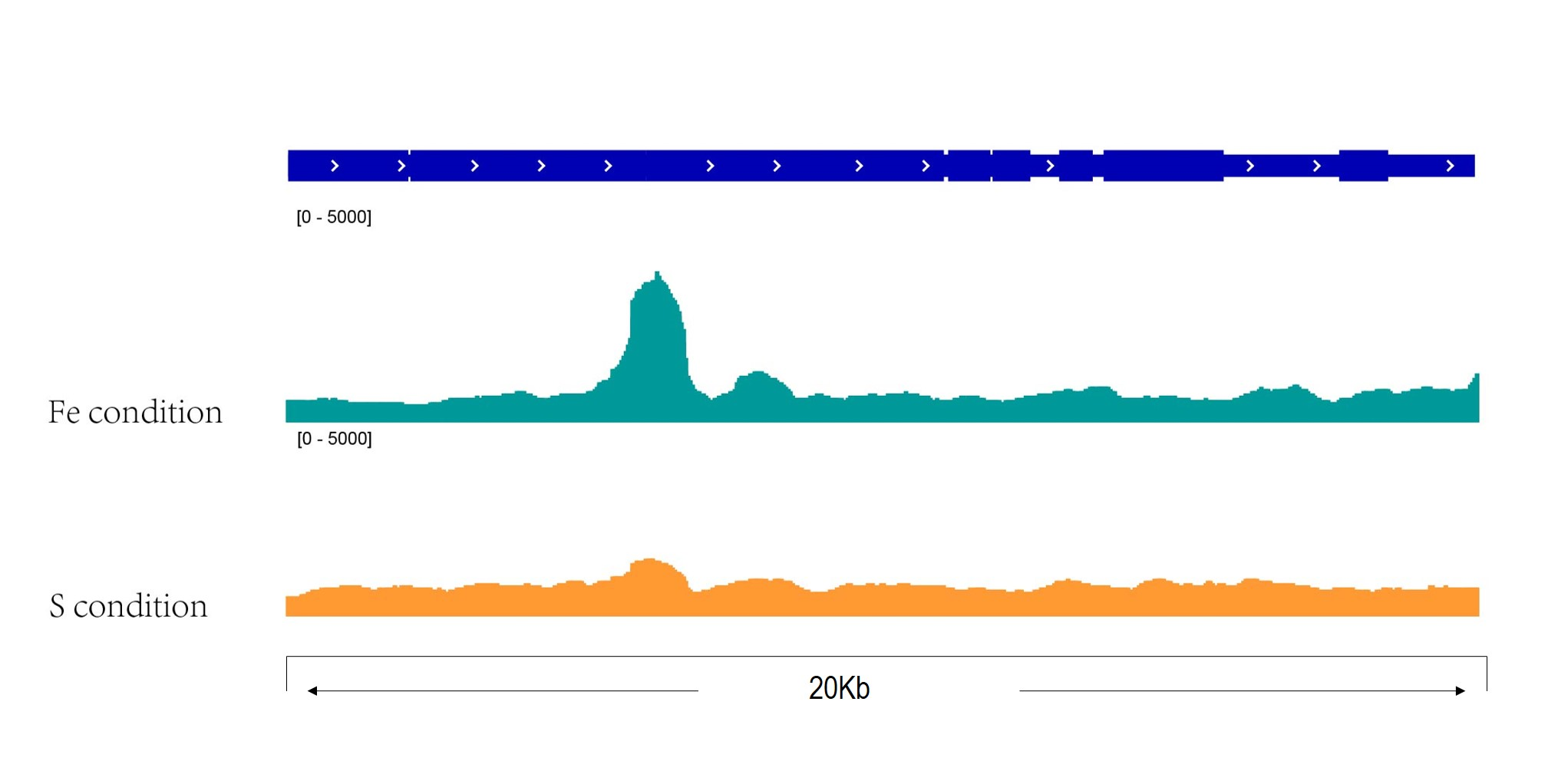

Supplement: S1 Fig — (TIF) [file pone.0298204.s003.tif]

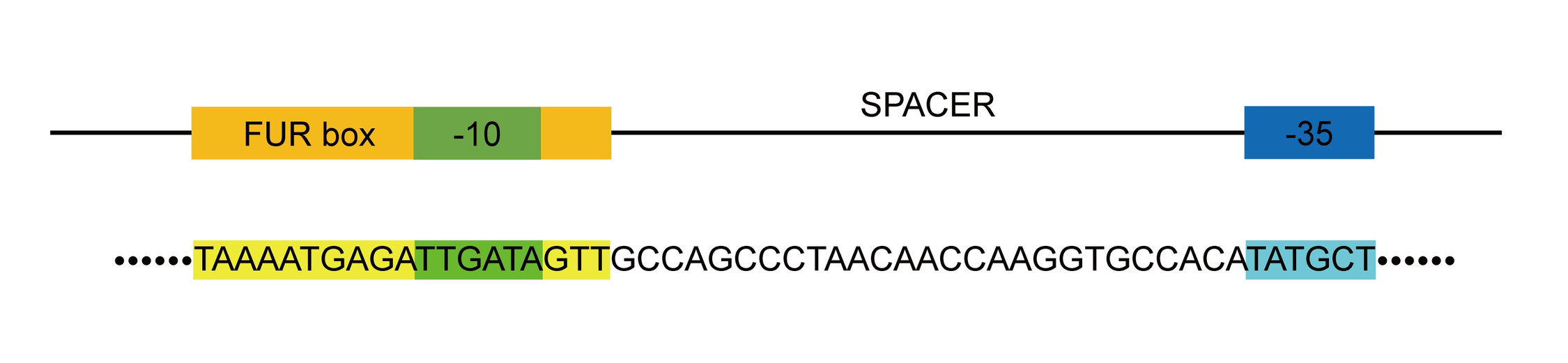

Supplement: S2 Fig — The -10 region, -35 region, and possible fur box are labeled with blue, green, and yellow boxes, respectively. (TIF) [file pone.0298204.s004.tif]
